# Diel transcriptional dynamics of a marine sponge and its microbiome in a natural environment

**DOI:** 10.1186/s42523-025-00510-z

**Published:** 2026-02-05

**Authors:** Gustavo A. Ramírez, Rinat Bar-Shalom, Tzipora Perez, Reut Efrati Epchtien, Andrea Furlan, Roberto Romeo, Michelle Gavagnin, Arkadiy I. Garber, Maya Lalzar, Laura Steindler

**Affiliations:** 1 Department of Marine Biology, Leon H. Charney School of Marine Sciences, University of Haifa, 199 Aba Khoushy Ave Mount Carmel, Haifa, Israel; 2 Department of Biological Sciences, California State University, 5151 State University Drive, Los Angeles, CA 90032 USA; 3 Istituto Nazionale di Oceanografia e di Geofisica Sperimentale (OGS), Trieste, Italy; 4 School of Life Science, Arizona State University, Tempe, AZ USA; 5 Bioinformatic Services Unit, Faculty of Natural Sciences, University of Haifa, Haifa, Israel

**Keywords:** Porifera, Microbiome, Animal-microbe interactions, Symbiosis, *Aplysina aerophoba*, Marine sponge, Diel cycle, Circadian, Holobiont, Cyanobacteria, Sulfur cycle, Glutathione, Taurine, Sulfite reduction, *Candidatus* Synechococcus spongiarum, Alphaproteobacteria

## Abstract

**Background:**

Marine sponges are ecologically critical animals that host diverse microbial communities, forming complex symbiotic systems crucial to nutrient cycling and ecosystem functioning in the ocean. While several studies have started to delineate the interactions between sponges and their microbiomes, whether and how these interactions are influenced by night and day cycles remains unclear, particularly in natural environments.

**Results:**

Here, we analyzed the in situ transcriptional changes of the demosponge species *Aplysina aerophoba* and its microbiome over a diel cycle, sampling four specimens each at four time points within a 24-hour period. At the global metatranscriptome level, microbiome activity clustered by animal subject, rather than time of sampling. However, diel transcriptional patterns were observed for a limited number of microbial genes, primarily involved in secondary metabolite biosynthesis and antibiotic efflux pumps. At the individual microbial lineage level, we identified diel patterns in cyanobacterial and alphaproteobacterial symbionts. Cyanobacterial symbionts exhibited canonical circadian regulation, with daytime expression of photosynthesis-related genes. At night upregulation of ammonium assimilation via glutamine synthetase coincided with induction of the oxidative pentose phosphate pathway and respiratory genes, indicating reliance on host-derived carbohydrates to generate ATP, NADPH, and the 2-oxoglutarate carbon backbone required for glutamate synthesis in the dark. This pattern contrasts with free-living cyanobacteria, where nitrogen assimilation is typically day-active and fueled by photosynthesis-derived energy and reducing power. The sponge host transcriptome displayed distinct diel regulation of key circadian genes (*cry2* and *PAR-bZIP*), in addition to daytime upregulation of genes involved in sulfur metabolism and oxidative stress defense, patterns mirrored by alphaproteobacterial symbionts that together contribute to glutathione-based detoxification (sponge Glo2, bacterial GstB) and H_2_S management (SQOR).

**Conclusion:**

Our study demonstrates that diel environmental fluctuations modulate the transcriptome of the sponge hosts and only select microbial lineages - primarily Cyanobacteria and heterotrophic Alphaproteobacteria – highlighting targeted rather than community-wide diel transcriptional responses within the holobiont. Together, these lineage-specific responses reveal mechanistic links between nitrogen assimilation, sulfur metabolism, and oxidative stress detoxification. These findings provide novel insights into the metabolic integration and functional stability of one of the earliest evolved animal-microbe symbiotic systems.

**Graphical Abstract:**

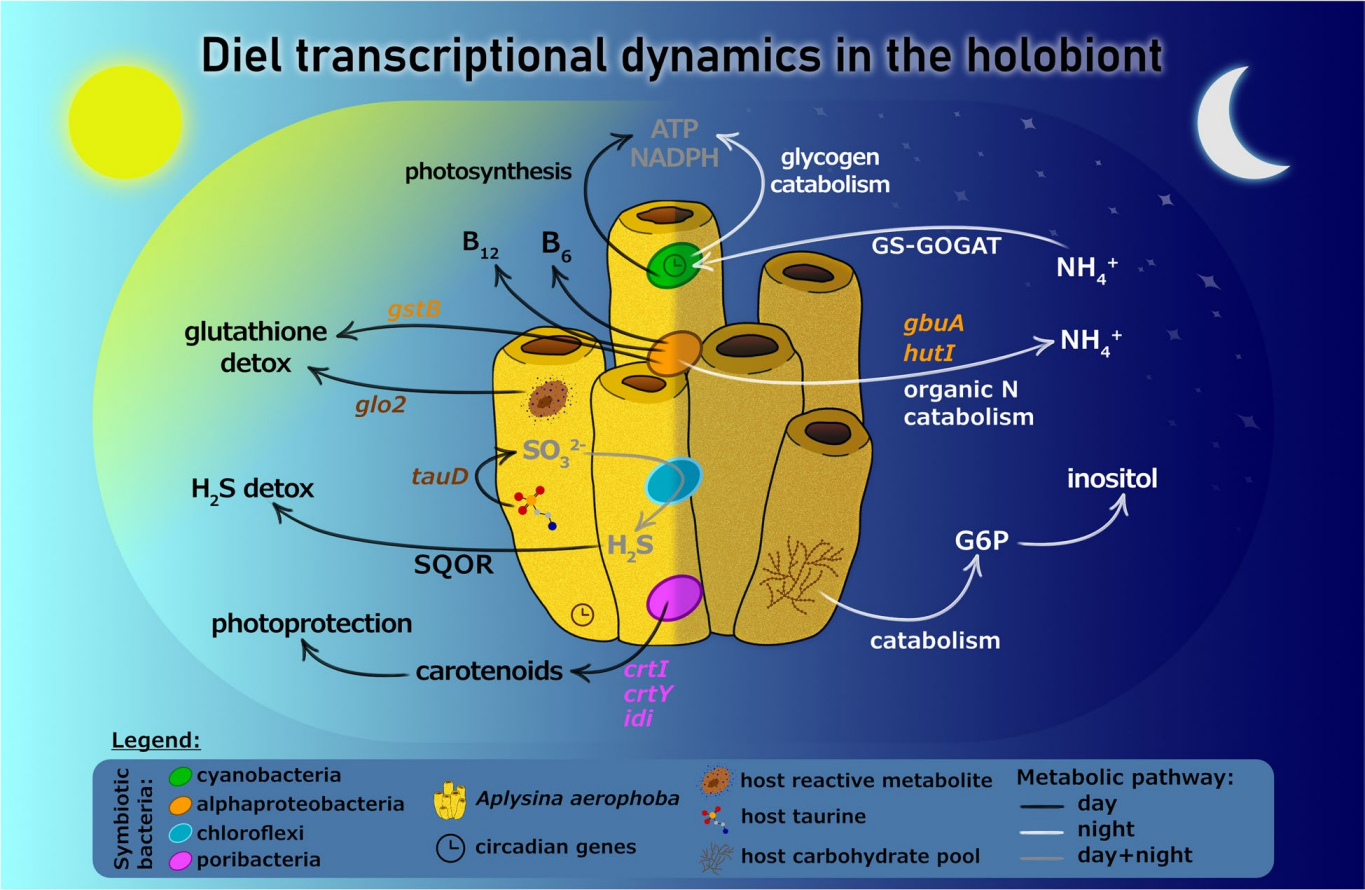

**Supplementary Information:**

The online version contains supplementary material available at 10.1186/s42523-025-00510-z.

## Background

Marine sponges are renowned for their capacity to filter vast volumes of seawater [1], playing a significant role in marine nutrient cycling and contributing to benthic-pelagic coupling. This process facilitates the transfer of carbon and nutrients from dissolved organic matter to higher trophic levels in the ocean [2]. Notably, these ecological functions are in part mediated by the diverse array of eukaryotic and prokaryotic microorganisms that are hosted by sponges, including zooxanthellae [3], bacteria and archaea [4, 5]. The host-specific and stable interactions with such microorganisms are believed to enhance the sponge’s physiological and ecological functions [4, 6]. For example, these symbiotic relationships are crucial for nutrient translocation [7], waste recycling [8], and reinforcing the sponge’s defense mechanisms through the production of secondary metabolites [9, 10]. The tight interactions between sponges and its microbiome require molecular mechanisms for host-microbe recognition [11] and likely entail coordinated metabolism within the holobiont. Metabolic coordination may arise from both sponge and symbionts responding to common environmental cues or from symbionts reacting to molecules produced by the host.

While studies have started to elucidate the mechanisms regulating sponge-microbiome interactions, one aspect that remains underexplored is how such interactions are modulated by diel (night-day) cycles. For instance, both sponges and symbionts may exhibit rhythmic daily behaviors triggered by diel-related environmental factors like light, nutrient availability, and oxidative stress generated during photosynthetically active periods. Alternatively, symbionts may respond to molecular signals from the sponge host or other symbionts, such as the diel influx of fixed carbon and oxygen resulting from photosynthesis by co-occurring photosymbionts. Although sponges have shown elements of circadian rhythm ([12, 13] and reviewed in [9, 10]), the potential for chronobiological patterns in the activities of sponge symbionts remains largely unexplored. In addition, diel rhythms of metabolism in free-living photosynthetic cyanobacteria [14] are well studied, but studies in host-associated cyanobacterial lineages are lacking. Beyond photosynthetic bacteria, marine heterotrophic bacterial assemblages also display diel oscillations, likely influenced by fluctuations in fixed carbon and oxygen resulting from phytoplankton activity [15–17], yet whether similar responses are present in sponge-associated lineages also remains unclear.

To better understand how diel cycles impact sponge-microbiome interactions, we examined the influence of the daily light-dark cycle on the transcriptional profile of photosynthetic and heterotrophic members of the bacterial community hosted by the sponge *Aplysina aerophoba*. Importantly, we performed these analyses in situ, sampling 4 sponge specimens from the Northern Adriatic in the Gulf of Trieste, each sampled 4 times in a 24 h period. Consistent with what is known for free-living cyanobacteria, we found that the transcriptome of symbiotic cyanobacteria displays diel periodicity. Interestingly, we also found diel cycling transcript profiles in three Alphaproteobacteria symbionts, with daytime upregulation of genes involved in sulfur metabolism and oxidative stress detoxification. In parallel, we observed the rhythmic expression of the sponge’s core circadian clock genes, as well as diel regulation of sponge genes linked to oxidative stress defense and detoxification of harmful compounds.

## Methods

### Sampling and sample preservation

Mediterranean *Aplysina aerophoba* specimens were sampled from the Northern Adriatic in the Gulf of Trieste (45°36.376, 13°43.1874), using SCUBA, within a few meters of each other. Four individual sponge specimens were separately sampled 4 times in a 24 h period (at 12:00 - Noon Day 1, 00:00 - midnight, 04:00 AM, and 12:00 - Noon Day 2). At each time point, approximately a 0.3 cm^3^ piece of tissue was excised from the lateral side of the chimney-like sponge structure. To avoid repeated sampling of the same region, each time point was taken from a different chimney within the same individual sponge. Red light-sticks were used to avoid exposing the sponge to light during night sampling. Immediately upon collection all samples were in situ preserved in RNA later solution using an underwater chamber as detailed elsewhere [18], and, once out of the water, kept on ice for a few hours prior to freezing and transport to shore-based storage at -80 °C.

### Preparation of libraries and metatranscriptomic sequencing

RNA was extracted using Allprep DNA/RNA mini kit (Qiagen, Germany). Briefly, each extraction was preformed using 30 mg of sponge sample placed in a Lysing Matrix E tube (MP Biomedicals, Santa Ana, CA) to which RLT buffer containing Reagent DX (Qiagen, Hilden, Germany) was added. Cells were disrupted using a TissueLyser II system (Qiagen, Germany) for 30 s at 30 Hz followed by 10 min centrifugation at maximum speed. All subsequent RNA extraction steps were performed according to the manufacturer’s protocol. SUPERase In (Life Technologies, USA) and TURBO DNA-free kit (Thermo Fisher Scientific, USA) were used for RNase inhibition and DNase treatments, respectively. RNA cleanup and concentration were done using RNeasy MiniElute kit (Qiagen, Germany). In order to achieve sufficient coverage of informative nonribosomal transcripts, rRNA was removed with RiboMinus Eukaryote System V2 kit (Ambion, Life Technologies, USA) with eukaryotic mouse-rat-human probes coupled with prokaryotic probes. ERCC RNA Spike-In Control mixes (Life Technologies, USA) were added to 5 µg of total RNA. RNA concentrations were measured using a Qubit 2.0 Fluorometer and RNA reagents (Thermo Fisher Scientific, USA), before and after rRNA depletion. In parallel, RNA integrity and purity were determined using a TapeStation 2200 system, applying the High sensitivity RNA Screen Tape assay (Agilent Technologies, USA), before and after rRNA depletion as well. Ultimately, 13ng of rRNA-depleted RNA were processed for cDNA libraries preparations using the Collibri stranded RNA library prep kit (Thermo Fisher Scientific, USA) according to the manufacturer’s protocol with the sole exception being that, following addition of the index codes, cDNA amplification was performed with 8 rather than the 9–11 recommended PCR cycles. The number of PCR cycles was optimized for our samples to reduce PCR bias. The libraries were quantified using Invitrogen Collibri Library Quantification Kit (Invitrogen, Thermo Fisher Scientific) according to the manufacturer’s guide using Real-Time qPCR. For pre-sequencing quality control (QC), 2 µl aliquots of each provided library were pooled. The resulting QC pool was size and concentration checked on an Agilent D1000 TapeStation system and a Qubit 2.0 fluorometer, respectively. The pool was adjusted to 1nM and loaded on an Illumina MiniSeq Mid Output flow cell at 1.5pM. After demultiplexing the percent of each library was used to calculate new volumes to use for constructing a normalized sequencing pool. This pool was also size and concentration checked, as described above, and subsequently normalized to 2nM. The normalized pool was run on an Illumina NextSeq High Output flowcell at 2.2pM.

### Sequence processing

Paired-end Illumina libraries were inspected for quality parameters and repetitive sequences using the FastQC software package. Adapter trimming was performed using the trim adapters bbduk script from BBMaps (https://sourceforge.net/projects/bbmap/). Trimmed paired-end files were interleaved for alignment against rRNA libraries using SortMeRNA [19]. Non-aligned reads were subsequently split into paired forward and reverse files for downstream analyses. A *de novo* co-assembly was performed using merged forward and reversed adapter trimmed and non-rRNA aligned sequences with rnaSPAdes v.3.14.1 [20]. Sequence counts at each step for all libraries, in addition to co-assembly summary statistics, are provided in Additional File 1: Table S1. Sequence data from the two noon time points were previously published [21], whereas the sequence data from the two nighttime time points (midnight and 4 AM) are newly reported in this study.

### ASV generation from 16S rRNA genes and transcripts

RNA, extracted in parallel with DNA, was subsequently reverse transcribed to cDNA. Both DNA and cDNA served as templates for PCR amplification of the 16S rRNA gene and transcript V4 region using the revised Earth Microbiome Primers [22]. The thermocycling program consisted of 94 °C for 3 min; 32 cycles of 94 °C for 45 s, 50 °C for 60 s, and 72 °C for 90 s; followed by 72 °C for 10 min and a 4 °C hold. Amplicons were sequenced on an Illumina MiSeq platform. DADA2 implemented in R was used for sequence processing [23]. Reads were trimmed with filterAndTrim(trimLeft = c(20, 20), maxEE = c(2, 2), phix = TRUE, multithread = TRUE, minLen = 120), followed by error modeling, dereplication, and read merging (mergePairs) with a minimum overlap of 120 bp. Amplicon sequence variants (ASVs) were taxonomically classified against the SILVA 132 database [24]. ASV count and taxonomy tables were integrated into a phyloseq [25] object for diversity and visualization analyses in RStudio.

### Read mapping

To assess coverage as a proxy for transcript abundance, quality-trimmed non-rRNA short reads were mapped to our *de novo* metatranscriptomic assembly and a public reference set of MAGs binned from *Aplysina aerophoba* in close geographical proximity to our study site [26] using Bowtie2 [27] and the following parameters: read counts were normalized to Transcripts per Million (TPM) per library, as suggested elsewhere [28], and all data was concatenated into read count tables for downstream statistical analyses.

### Phylogenomic tree

A phylogenomic tree was generated for *A. aerophoba* derived MAGs using the GToTree package [29]. Briefly, 37 publicly available MAGs [26] were used as the input and were run against a GToTree’s “Bacteria” HMM collection of single copy genes within this domain resulting in a concatenated protein alignment constructed using Muscle [30] and trimmed with TrimAl [31]. The tree was constructed in FastTree2 [32] and visualized using FigTree (https://github.com/rambaut/figtree).

### Differential expression – microbes

Differential expression testing was implemented using DESeqs2 [33]. Briefly, Wald tests for significant (Pval = 0.05) multiplicative changes in PET expression across comparison models (e.g.,: dark vs. light at collection time, specimen *x* vs. specimen *y*, etc.) were performed on model-specific geometric mean scale normalized read counts [34]. PETs with significant differences across the model comparisons were reported by plotting either Log2 fold change and/or individual *recA*-normalized TPM summaries.

### Canonical correspondence analysis

Dimensionality reduction of TPM-normalized PET count tables stored as S4 objects was performed using the ordinate() function and “CCA” method [35] available in the R package phyloseq [25]. Resultant ordinations were plotted using the ggplot2 R package [36], also implemented in phyloseq via the plot_ordination() function.

### ORF calling, annotation, and targeted gene analyses

Open reading frame (ORF) identification and, subsequently, prokaryote predicted protein product annotations were performed with Prodigal v2.6.1 [37] implemented in Prokka [38]. Selected ORFs were also aligned against the NCBI non-redundant (nr) database (accessed in April 2021) using BLASTp for closest homologue taxonomy and functional annotation supplementation. Targeted single gene homologue searches within our data were also performed using BLASTp 2.2.30+ (*E*-value threshold = 1E − 30, identity = 50%) against predicted protein sequences inferred from (i) metatranscriptomic assemblies, (ii) unbinned metagenomic contigs, and (iii) MAGs. Lastly, we developed a python executable tool implementing pHMMs against circadian regulatory genes called *CircGenie* and used it to search for circadian gene homologues in the MAGs. Additional information on *CircGenie* development and implementation is found in the extended materials and methods section in the supplemental.

### Reverse-Transcriptase qPCR

Custom primer sets designed against the Cyanobacterial genes *kaiC*, *psbA*,* and recA* (the latter used as normalizing gene) were used for transcript quantification from each of our 16 metatranscriptomic libraries. Briefly, total cDNA libraries were produced using the High-Capacity cDNA Reverse Transcription Kit (Applied Biosystems) following the manufacturer’s protocol. Subsequently, target gene-optimized quantitative PCR cycling programs were implemented in a StepOne™ software v2.3 qPCR cycler using the Applied Biosystems SYBR green qPCR kit following the manufacturer’s instructions. Quantification was performed by endpoint comparisons against target specific standards covering a range of 10^2^ to 10^8^ gene copy numbers using the Quantitation-Standard Curve software package. The R^2^ value for all standard curves was > 0.99 with estimated minimum amplification efficiencies of 100%. The following primer sets were used for gene-targeted analyses: Ssp_kaiC_F: 5’-CTTTGTTCTGGACGCCTCTC-3’, Ssp_kaiC-R: 5’- CACCCGTTTGGCTTTGTACT-3’; Ssp_ psbA_F:5’-CAACCTCAACGGCTTCAACT-3’, Ssp_psbA_R: 5’- ATCACTTCCATGCCCAGACT-3’; Ssp_recA_F: 5’- GACATTCGCCGTATCCAAAC-3’, Ssp_recA_R: 5’- CACCTTGTTCTTGGCCACTT-3’.

### Host-metatranscriptome analysis

The reference genome data for *A. aerophoba*, including genome sequences, gene transfer format (GTF) files, and protein sequences, were retrieved from the Ensembl Aquatic Symbiosis Genomics project (https://projects.ensembl.org/asg/) [39]. The genome contains 44,719 predicted proteins. Gene annotation was performed using DIAMOND blastp [40] against the UniProt/SwissProt database [41], resulting in the annotation of 17,589 genes​. RNA sequencing reads underwent quality assessment using FastQC. Low-quality reads (quality < 20, minimum length = 100 bp) were removed using BBDuk: Adapter/Quality Trimming and Filtering​. High-quality reads were aligned to the reference genome using RNA-STAR (v2.7.11b), an ultrafast spliced aligner for RNA sequencing reads [42]. Total number of reads from each sample that were mapped to genes, are shown in Additional File 1: Table S2.

Gene-level expression was quantified as the number of reads mapped per gene for each sample​. To identify differentially expressed genes, a filtering step was applied, retaining genes expressed in at least six samples. After filtering, 15,322 genes were included in the analysis. DESeq2 (v1.42.1) was used for statistical analysis of differential gene expression, with normalization performed using the Median Ratio Normalization (MRN) method [43]. Boxplots were generated using ggplot2 (v3.5.1). For differentially expressed genes that did not have a Swissprot annotation, functional domain annotation was performed using InterProScan (version 5.73–104.0) [44] to identify conserved protein domains and functional motifs.

Non-metric multidimensional scaling (NMDS) was performed to assess sample clustering based on normalized read counts derived from DESeq2 outputs. Visualization was conducted using the ggplot2 package in R.

## Results and discussion

### Sponge microbial community stability

To characterize the impact of a diel cycle in the transcriptional response of a sponge holobiont, we sampled 4 mediterranean *A. aerophoba* specimens separately at 4 times in a 24 h period (at 12:00 - Noon Day1, 00:00 - midnight, 04:00 AM, and 12:00 - Noon Day2) (see Methods for details). To ensure that observed transcriptional changes reflected shifts in microbial activity rather than alterations in microbial community composition, we assessed microbial community structure across time by generating 16S rRNA gene libraries for each sampled metatranscriptome. Microbial community structure based on genomic (g)DNA was statistically indistinguishable between different individual specimens as well as across the four time points (PERMANOVA, Pval > 0.05). This confirmation is critical, as our metatranscriptomic analyses assume that temporal variation in gene expression reflects changes in microbial activity rather than shifts in community composition.

We generated 16S rRNA gene amplicon sequence variant (ASV) libraries using both gDNA and complementary (c)DNA as amplification template. cDNA libraries are considered indicators of the transcriptionally active fractions of microbial communities. Our results show that the overall microbial community composition of the sampled sponges is similar to the patterns previously reported for *A. aerophoba* [26, 45, 46]. In brief, Proteobacteria (Pseudomonadota), Chloroflexota, Cyanobacteriota, Acidobacteriota, and *Candidatus* Poribacteriota (formerly referred to as Poribacteria) dominate all samples and, both at the phylum and class level, few qualitative differences are observed between gDNA and cDNA in predicted community structure (Supplementary Fig. S1).

**Fig. 1:**
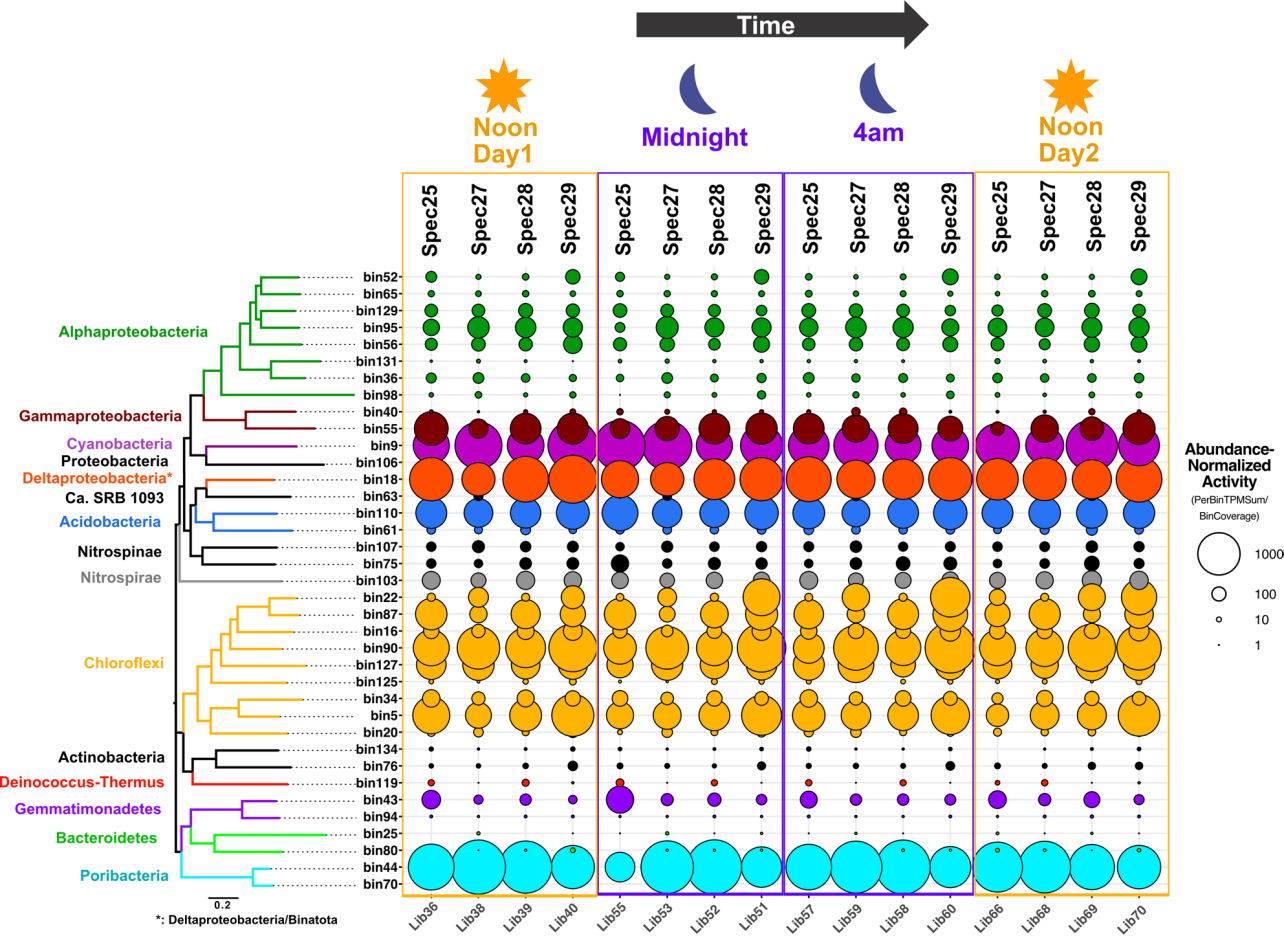
Global microbial gene expression levels of dominant lineages do not show diel periodicity. Metagenome assembled genome (MAG)-based metatranscriptomic read recruitment analysis of the gene expression levels of the 37 dominant microbial communities associated with 4 mediterranean *Aplysina aerophoba* specimens (Spec25, Spec27, Spec28 and Spec29). Each sponge was sampled 4 times in a 24h period (at 12:00 - Noon Day1, 00:00 - Midnight, 04:00 AM, and 12:00 - Noon Day2). In the balloon plot, circle size represents the bin-specific sum of transcripts (transcripts per million, TPM, normalized by metagenomic coverage of the bin) for each time-stamped (Noon Day1, Midnight, 4 AM, Noon Day2) library (Lib). Circle colors represent taxonomic assignments of phylogenomic clusters. *Bin18, an unclassified Deltaproteobacteria member based on NCBI taxonomy [26], has been recently proposed as a member of the Candidate Phylum Binatota [48]

A metatranscriptomic read recruitment analysis against Metagenome-Assembled Genomes (MAGs) revealed the abundance-normalized activity of 37 *A. aerophoba*-associated MAGs, including members of Proteobacteria (Pseudomonadota), Cyanobacteriota, Acidobacteriota, Chloroflexota, and Candidatus Poribacteriota (Fig. 1). Certain MAGs affiliated with Deltaproteobacteria, Cyanobacteriota, *Candidatus* Poribacteriota, Chloroflexota and Gammaproteobacteria are among the most active symbionts, in agreement with 16S rRNA amplicon data derived from cDNA (Supplementary Fig. S1). Similar to microbial community composition, transcriptional activity of these dominant lineages exhibited a highly conserved pattern among individual sponge subjects. This stability in abundance-normalized gene expression – interpreted here as relative activity levels – was independent of phylogenetic relatedness. For example, within the same phylum, we observed substantial variance in MAG-specific transcriptional activity. In Chloroflexota, a phylum widely associated with sponges [47], different lineages consistently differed in their activity levels (e.g. bin90 versus bin125), and this pattern remained stable across individual animal subjects. Similarly, among Acidobacteriota, bins 61 and 110 exhibited persistently low and high activity levels respectively, across independent animal subjects.

At this broad global transcriptional analysis level, diel periodicity was not detected across the 37 analyzed lineages. Instead, MAG-centric activity trends remained stable and were specific to each microbial lineage across all individual specimens and time points (Fig. 1). These findings suggest that the structure and activity of the *A. aerophoba* microbiome are not random but rather highly conserved across sponge subjects.

### Genes differentially expressed between day and night samples

To identify microbial genes differentially expressed between day and night samples, we performed an analysis comparing the combined day samples (Noon Day1 and Noon Day2) against the combined night samples (midnight and 4 AM). This revealed a few (*n* = 12) significantly (Wald Test, Pval < 0.01) differentially expressed protein encoding transcripts (PETs) (Additional File 1: Table S3). Different model comparisons, such as (i) combined noon samples from days 1 and 2 versus 4 AM alone and (ii) combined noon samples from days 1 and 2 versus midnight alone, using identical test parameters (Wald Test, Pval < 0.01), yielded more differentially enriched transcripts: a total of forty-seven non-redundant, day-time enriched PETs largely classified as hypothetical proteins (Additional File 1: Table S4). This limited number of differentially expressed genes was unexpected, since light exposure has been previously linked to the increased microbial production of bioactive metabolites in the light exposed ectosome of *A. aerophoba* [49]. However, at the global metatranscriptomic scale, individual gene expression changes may be statistically masked by the overall stability of microbiome activity (Fig. 1).

**Fig. 2:**
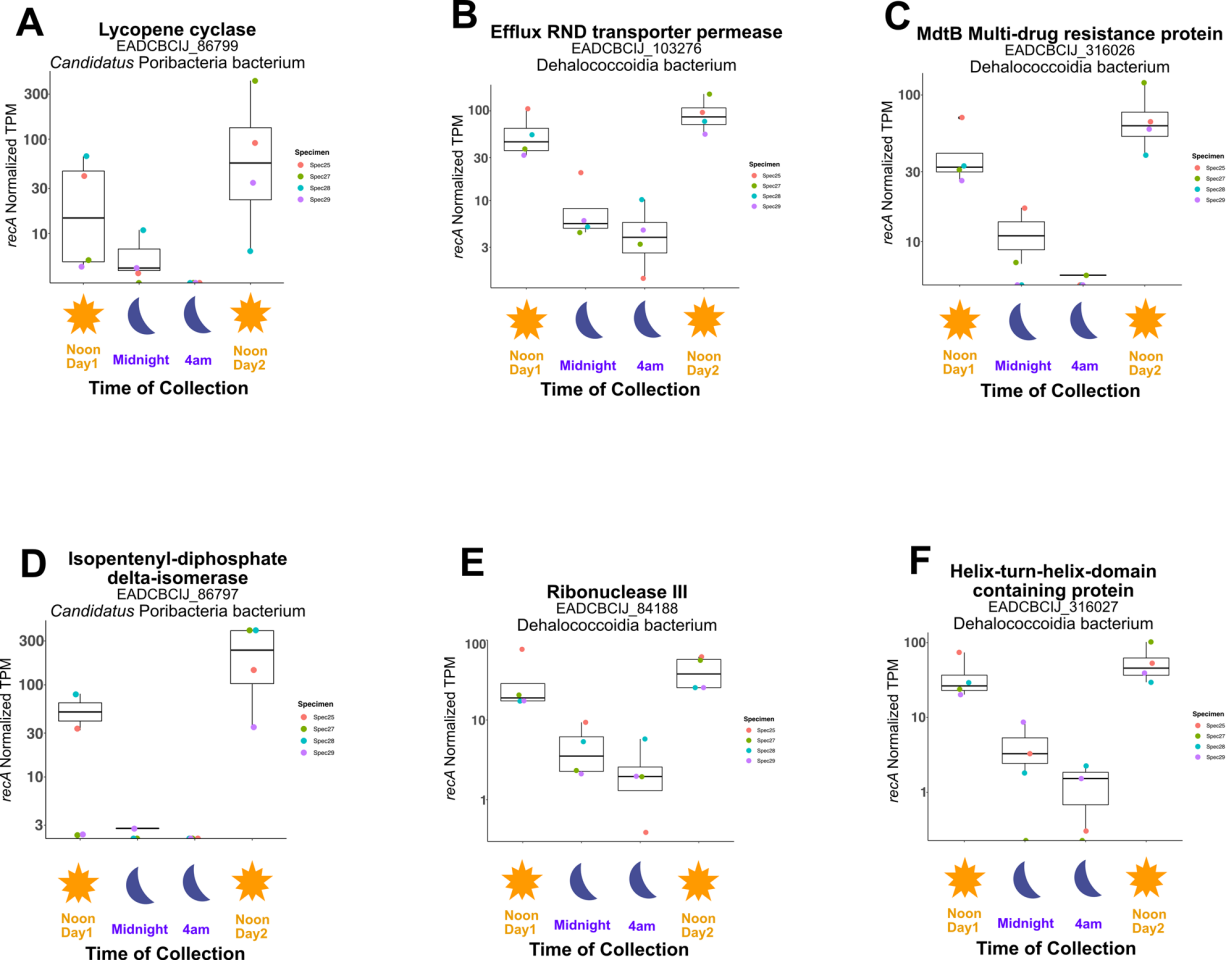
Microbial protein encoding transcripts (PETs) displaying diel periodicity. Data points show *recA* normalized transcripts per million (TPM) for selected transcripts. Data is summarized with boxplot quartiles, showing values for biological replicates, color coded as a function of specimen source for each timepoint (Noon Day1, Midnight, 4 AM, Noon Day2). (**A**) Lycopene cyclase, (**B**) Efflux RND transporter permease, (**C**) MdtB multidrug resistance protein, (**D**) Isopentenyl-diphosphate delta-isomerase, (**E**) Ribonuclease III, and (**F**) Dehalococcoidial hypothetical protein. Additional differentially expressed genes in the day vs. night are listed Additional File 1: Table S2

Despite the overall stability in microbiome activity, we identified PETs that exhibited diel periodicity with peaks in transcription during daylight hours (Fig. 2, Additional File 1: Tables S3 and S4). These included PETs associated with beta-carotene biosynthesis, antimicrobial resistance, and RNA decay/maturation (Fig. 2). Lycopene cyclase and isopentenyl-diphosphate delta-isomerase PETs, belonging to a member of the *Candidatus* Poribacteriota phylum, are both involved in carotenoid biosynthesis. High daytime levels of carotenoid biosynthesis is consistent with their proposed role in photoprotection of sessile marine invertebrates [50]. Compounds in this pigment family specifically protect against UV radiation that bathes benthic habitats as deep as 20 m [51] and are non-enzymatic antioxidants that neutralize reactive oxygen species (ROS) resulting from photocatalysis [52]. Similarly, PETs affiliated with Chloroflexota (Dehalococcoidia), and Poribacteriota, involved in multidrug efflux pumps, RNA maturation, TCA and pentose phosphate pathway activity (ATP synthase subunits, fumarate hydratase, malate dehydrogenase, 6-phosphogluconate dehydrogenase), also displayed diel variation with transcriptional peaks during daylight hours (Additional File 1: Table S4). The higher abundance of Dehalococcoidia-associated Ribonuclease III transcripts during daylight hours suggests increased protein turnover and regulatory activity [53]. The higher abundance of PETs associated with Resistance-Nodulation-Division (RND) efflux pumps - recently identified in the proteome of an organohalide-respiring symbiont of *A. aerophoba* [54] - suggests a rhythmic pattern in detoxification and antibiotic resistance mechanisms performed by Dehalococcoidia. This pattern may reflect a net increase in antimicrobial production by the sponge microbiome during daylight hours. Photosynthesis (RuBisCO chains, photolyase, C-phycoerythrin subunits) and cell division activity (FtsZ) associated PETs of cyanobacterial provenance were expectedly differentially enriched in the day sample (Additional File 1: Table S4).

### Within-host changes in microbiome activity profiles

**Fig. 3:**
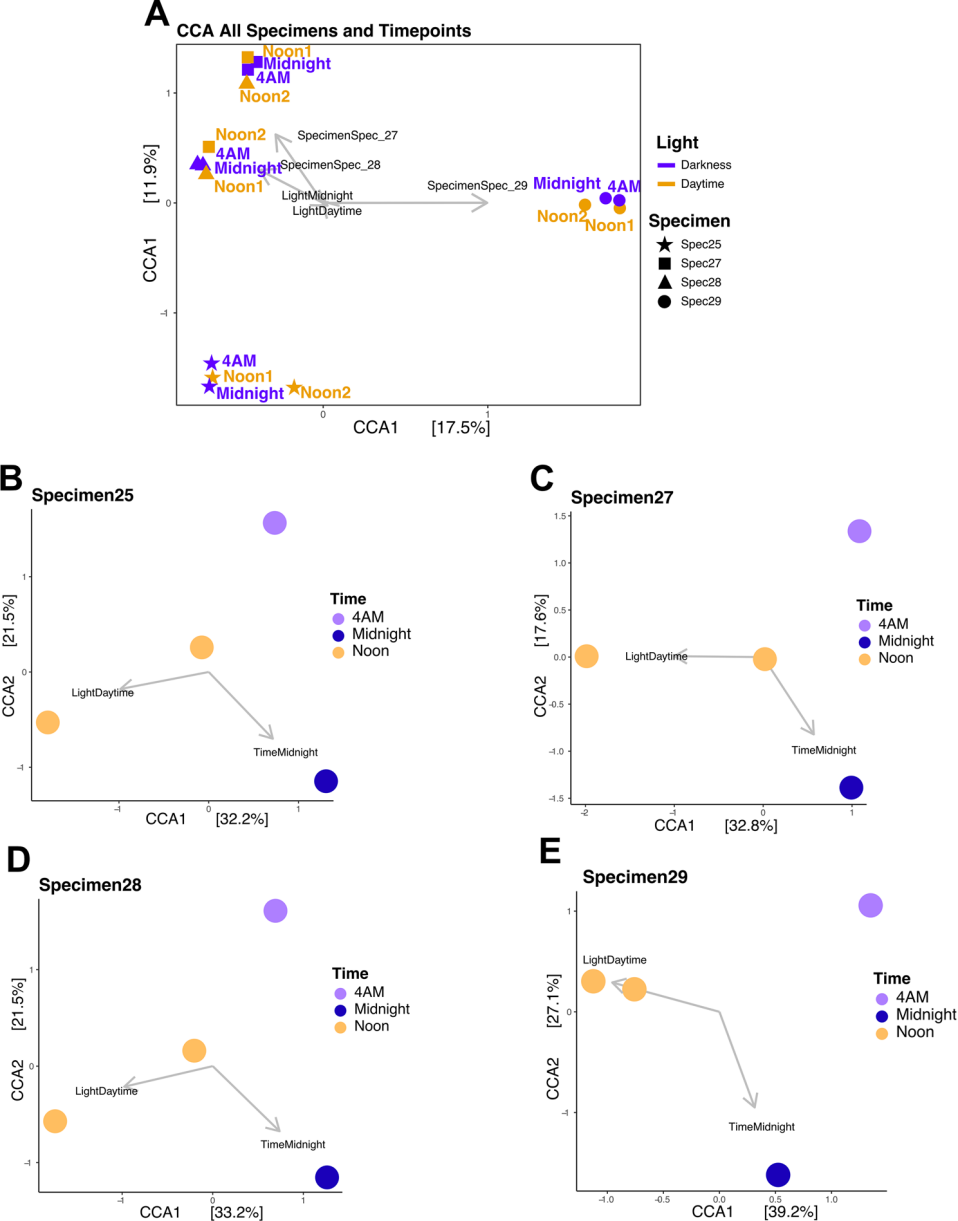
Global and specimen-based ordination structuring of microbial metatranscriptomes. Canonical correspondence analyses of metatranscriptome profiles for combined (**A**) and individual (**B**-**E**) sponge specimens. **A**) Colors represent light/dark conditions at the time of collection, shapes designate specimen individual, colored labels show exact collection time, and vector/labels depict the strength of each variable in observed clustering. **B**-**E**) Colors represent time of collection, and vector/labels depict the strength of each variable observed clustering for each of our 4 sponge subjects, each sampled 4 times in a 24 h period (Noon Day1, Midnight, 4 AM, and Noon Day2)

Overall, metatranscriptomic profiles cluster primarily by individual sponge subjects rather than by time of collection (Fig. 3A). This suggests that the specific host animal exerts a stronger influence on global transcriptional patterns than diel cycles. However, when analyzing microbiome activity within individual sponges, time of collection emerges as a secondary driver of transcription differences (Fig. 3B-E). Specifically, ordination clustering based on daytime (combined Noon Day1 and Noon Day2) versus nighttime (combined midnight and 4 AM) samples reveals subtle but consistent diel patterns within each sponge subject (Fig. 3B-E). This effect is most pronounced in Specimen 29, where Axis 1, separating light from dark samples, accounts for 39.2% of the variance. These results indicate that despite the high level of microbial community structure similarity across different sponge subjects (Fig. 1, Supplementary Fig. S1), microbiome transcriptional activity profiles are shaped by host-specific factors. While diel periodicity does not dominate global transcriptional patterns, the observed within-host clustering suggests that light-responsive lineages are indeed present but are limited to a subset of the microbiome and, consequently, the metatranscriptome (see below).

### Light-driven activity profiles in Cyanobacteria, Alphaproteobacteria, Poribacteriota, and Chloroflexi

**Fig. 4:**
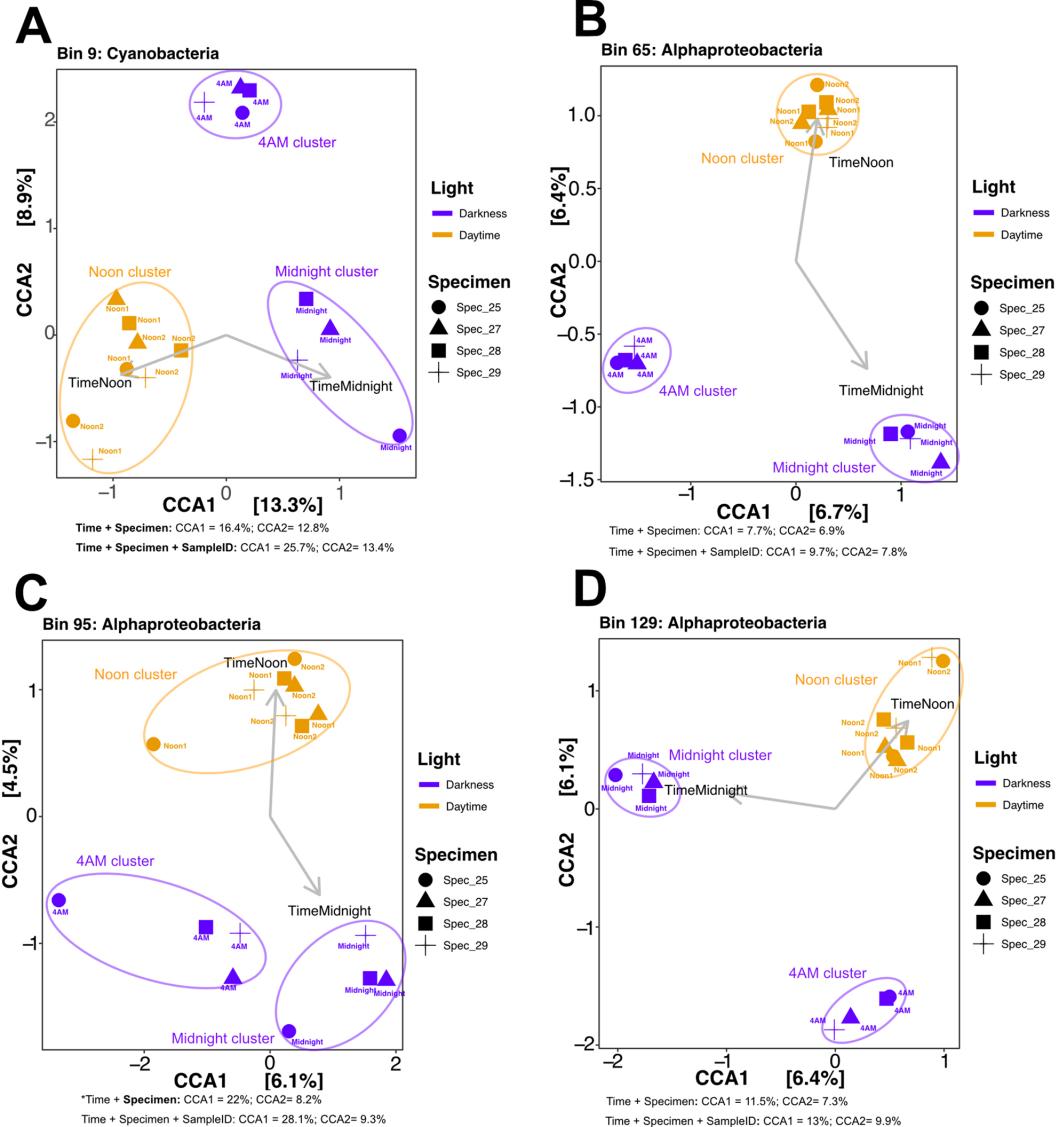
Cyanobacteria and Alphaproteobacteria show light-driven activity profiles. Canonical correspondence analyses of lineage-specific MAGs [bins 9, 65, 95, and 129 (panels **A**-**D**, respectively)] based on transcriptional profiles for all specimens and collection times. Colors represent light conditions at the time of collection, shapes designate specimen source, colored labels depict exact collection time, and vector/labels depict the strength of each variable in the observed clustering

We then further explored the influence of light presence or absence on the transcriptional profiles of individual microbiome lineages (MAGs). Among the 37 high-completion MAGs analyzed (Fig. 1), only cyanobacterial and alphaproteobacterial MAGs exhibited light-related activity patterns (Fig. 4). Notably, both are highly abundant and active lineages in our samples (Supplementary Fig. S1) and in sponges globally [46].

As expected, the transcriptional profile of Cyanobacteria (bin9), a photosynthetic bacterium, clustered according to time of collection (Fig. 4A). Cyanobacteria bin9 belongs to the *Candidatus* Synechococcus spongiarum clade, a geographically widespread, sponge-specific lineage found in diverse sponge species and known to contain a fully entrained circadian clock [55–58]. More unexpectedly, light also influenced the clustering of alphaproteobacterial bins 65, 95, and 129 (Fig. 4B-D), although to a lesser extent than for Cyanobacteria. Light accounted for a larger amount of the variance in the cyanobacterial lineage ordination (13.3% of the variance along CCA axis1; Fig. 4A). By contrast, for the Alphaproteobacteria, light explained a smaller fraction of the variance (6.4, 4.5, and 6.1% along CCA axis2 for bins 65, 95, and 129 respectively; Fig. 4B, C, and D).

No light-related CCA clustering was observed for the transcriptional profiles of other dominant lineages (e.g.: Chloroflexi and Poribacteriota MAGs; Supplementary Fig. S2A-D). Nevertheless, the global metatranscriptomic co-assembly revealed differentially expressed transcripts annotated as Chloroflexi and Poribacteriota (Fig. 2, Additional File 1: Table S3, S4). To further investigate these patterns, we performed a targeted differential expression analysis within individual MAGs for both lineages. Chloroflexi MAGs (bins 5, 16, 20, 22, 34, 87, 90, 125, 127) showed no differentially expressed genes across light-dark models (data not shown), suggesting that Chloroflexi maintain constitutive transcriptional activity throughout the 24-hour sampling period. In contrast, Poribacteriota MAGs exhibited light-responsive expression. Specifically, bin44 displayed up-regulation of transcripts encoding phytoene desaturase, lycopene cyclase, isopentyl-diphosphate delta-isomerase, and TonB family transporters, alongside additional hypothetical proteins, under light conditions (Supplementary Fig. S3, Additional File 1: Table S5). These findings indicate that certain Poribacteriota members participate in photoprotective pigment biosynthesis and active acquisition of complex organic substrates in response to light.

### Differential expression of cyanobacterial genes with diel periodicity

To identify the genes driving light-associated clustering, we performed differential gene expression analyses (Wald Test, Pval < 0.01) on the cyanobacteria MAG (bin9) that exhibited diel activity (Supplementary Fig. S4B). In Cyanobacteria bin9, eighty-three non-redundant genes were differentially expressed between the daylight-exposed samples (combined Noon Day1 and Noon Day2), and the midnight samples and the 4 AM samples independently (Supplementary Fig. S4B). Importantly, no significant differences were observed between Noon Day1 and Noon Day2.

**Fig. 5:**
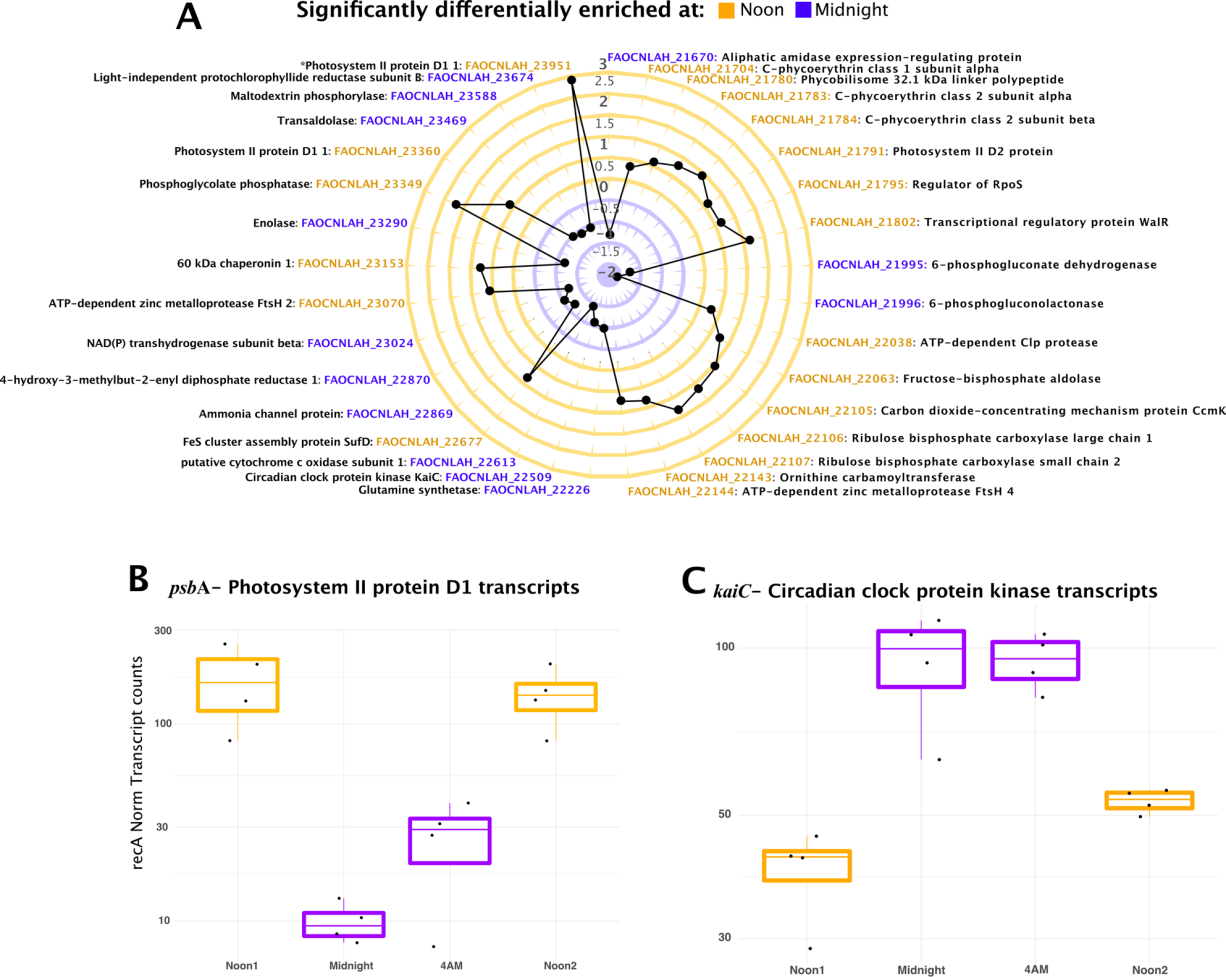
Differentially expressed Cyanobacterial genes between noon and midnight samples. (**A**) Radar plot depicting genes that were significantly enriched in midnight (purple gene names, inner purple circle with negative Log2Fold values) and noon (yellow gene names, outer yellow circle with positive Log2Fold values) samples based on metatranscriptomics data. (**B**) Summary of *recA* normalized *psbA* transcript counts at each collection timepoint based on qPCR data analysis. (**C**) Summary of *recA* normalized *kaiC* transcript counts at each collection timepoint based on qPCR data analysis

Annotated PETs that were noon-enriched and midnight-depleted include key photosynthetic components such as Photosystem II proteins (*psb*), C-phycoerythrin class 1 (*cpe*), phosphoglycolate phosphatase (*pep*), fructose-bisphosphate aldolase (*fba)*, and ribulose bisphosphate carboxylase (*rbc*) (Fig. 5A, Additional File 1: Table S6). Additionally, FeS cluster protein (*sufD)*, which is involved in oxidative repair, and carbon dioxide concentrating protein (*ccmK*), which enhances Rubisco efficiency, were both upregulated during the day. Conversely, annotated PETs that were enriched at midnight and depleted at noon include maltodextrin phosphorylase (*mal*P), enolase (*eno*), transaldolase (TALDO), and phosphogluconolactonase (*pgl)*, all of which are involved in carbohydrate metabolism and regulation (Fig. 5A). This pattern is consistent with observations in free-living cyanobacteria, where the absence of photosynthesis at night limits the availability of reducing power (NADPH). To compensate, cells degrade glucose accumulated during the day, via the oxidative pentose phosphate pathway (OPPP), thereby generating NADPH. This reducing power is essential for the activity of multiple enzymes, including those involved in detoxifying reactive oxygen species (ROS) produced during daylight [59]. Because both ATP and NADPH are diminished in the dark, the observed upregulation at night of cytochrome *c* oxidase, which supports ATP production through respiration, and NADP(H) transhydrogenase (*pntB*) which generates NADPH from NADH (Fig. 5A), likely mitigates this reduction in energy and redox capacity.

Intriguingly, we also detected nighttime upregulation of glutamine synthetase (*glnA*) and an ammonia channel in *Ca*. S. spongiarum (Fig. 5A), a pattern that contrasts with free-living cyanobacteria. In the latter, nocturnal decreases in 2-oxoglutarate, the carbon backbone required for ammonia assimilation via the GS/GOGAT cycle, lead to low cellular C/N ratios and consequent downregulation of nitrogen assimilation genes, including *glnA* [60, 61]. Taken together, the enrichment of OPPP transcripts at night in symbiotic cyanobacteria suggests that these organisms may receive host-derived carbohydrates during the dark period. Such inputs would fuel respiration, as supported by the upregulation of cytochrome *c* oxidase and *pntB*, enhancing oxidative phosphorylation and redox balancing. The resulting ATP and reducing power, generated in the absence of photosynthesis, may enable the symbiont to sustain nitrogen assimilation at night, thereby producing amino acids that could also be supplied to the sponge host.

Notably, transcripts of *kaiC*, a well-characterized circadian clock regulator known to peak at night in Cyanobacteria [62], were significantly upregulated at midnight compared to noon samples (Fig. 5A, FAOCNLAH_22509).

To validate these diel transcription patterns independently of metatranscriptomic analyses, we performed reverse-transcription quantitative PCR (RT qPCR) using custom primers targeting key photosynthesis and circadian clock-related genes. The results confirmed our metatranscriptomic findings, with *psbA* transcripts enriched during the day and *kaiC* transcripts peaking at night (Fig. 5B, C).

To further investigate the presence of circadian core regulator gene homologs across all dominant MAGs generated from this sponge species we used Pfam-derived profile Hidden Markov Models (pHMMs), implemented via *CircGenie*, a custom software tool now made public through this publication (see Extended Materials and Methods section of the SI). Unsurprisingly, circadian regulator gene homologs were only found in cyanobacterial bin9 (Supplementary Fig. S5).

Collectively, our findings suggest that, similar to free-living cyanobacteria - where KaiA, KaiB, and KaiC circadian clock system has been described as a regulator of global gene expression [62, 63], cell division [64], and competence [65] - sponge-associated cyanobacteria also exhibit light/dark core circadian oscillator activity *in situ.* Nevertheless, nitrogen assimilation may be largely responsive to metabolic cues such as C/N balance, which can differ in symbionts compared to free-living Cyanobacteria, potentially explaining why ammonia assimilation is upregulated during day in free-living Cyanobacteria but at night in sponge-associated symbionts.

### Differential expression of Alphaproteobacterial genes with diel periodicity

To resolve the temporal transcriptional patterns observed for the Alphaproteobacteria (Fig. 4B-D), we compared noon-collected samples separately with midnight- and 4 AM-collected samples. This analysis revealed 514, 34, and 51 differentially expressed genes between noon and midnight and 494, 31, and 48 differentially expressed genes between noon and 4 AM, in alphaproteobacterial bins 65, 95, and 129, respectively (Supplementary Fig. S6, Additional File 1: Table S7). Between 29% and 44% of the genes differentially expressed between light (combined Noon Day1 and Noon Day2) and dark (midnight or 4 AM) conditions were enriched in the daylight samples (Supplementary Fig. S6A). The majority of genes enriched in darkness were specific to either midnight or 4 AM, with only ~ 14% of differentially expressed genes shared between both dark time points. The majority of differentially expressed alphaproteobacterial genes were annotated as hypothetical proteins (Supplementary Fig. S6B). Overall, among genes with functional annotations, we observed daytime enrichment of genes involved in radiation repair, oxidative stress response, vitamin metabolism, nitrogen cycling, and detoxification (Supplementary Fig. S6C-E). For example, genes associated with nucleic acid repair – such as Exodeoxyribonuclease III, Acyl CoA Dehydrogenase AidB, and 60 kDa Chaperonin I – were commonly enriched in daylight (Supplementary Fig. S6C). These genes are typically linked to UV-induced DNA and protein damage repair or prevention [66–68], explaining their strong diurnal upregulation. Another major category of day-associated activity was B-vitamin biosynthesis (particularly B1, B2, B3, and B12) (Additional File 1: Table S7). Diurnal upregulation of vitamin B12-related genes, specifically adenosylcobinamide-GDP ribazoletransferase (*cobS*) and nicotinate nucleotide dimethylbenzimidazole phosphoribosyltransferase (*cobT*), which encode enzymes for the final steps of cobalamin biosynthesis [69], was consistently observed across all three alphaproteobacterial lineages (Supplementary Fig. S6D). These findings underscore the role of cobalamin provisioning in host-microbe interactions, its potential contribution to sponge fitness [70], and is further discussed below in the section dedicated to sulfur cycling. Additionally, we observed daytime upregulation of Argininosuccinate synthase, an enzyme involved in arginine metabolism and nitrogenous waste removal [71], and genes related to isonitrile hydratase [72] and aromatic compound degradation (4-hydroxybenzoyl-CoA reductase subunit beta) [73] (Supplementary Fig. S6E). Symbiont-produced arginine, which we found to be upregulated during daylight, can serve as a precursor for nitric oxide (NO), a key regulator of developmental processes in many marine invertebrates, including demosponges [74]. This diurnal pattern suggests that light-driven metabolic coordination between host and symbiont may influence signaling pathways and developmental physiology, pointing to a broader role for synchronized metabolic cycling in sponge holobionts. Interestingly, genes involved in carnitine metabolism – a process that utilizes sponge-derived cellular detritus – were also upregulated during the day (Additional File 1: Table S7). As per the differentially expressed genes of hypothetical function, domain-specific analysis using InterPro revealed that many contain transmembrane features, DUFs, transcription regulatory motifs, or RNA-processing signatures (Additional File 1: Table S8). These features provide functional clues for interpreting their differential expression and suggest potential roles in membrane remodeling, gene regulation, and stress response.

Collectively, our analyses suggest that the light-driven activity of the heterotrophic Alphaproteobacteria associated with sponges likely reflects indirect responses to light-induced changes in environmental conditions, the microbial community or the host sponge, rather than direct light sensing by these microbes. Some light-enriched functions, such as waste removal, antibiotic production, and aromatic hydrocarbon degradation may be driven by metabolites released by other, perhaps light responding, members of the sponge microbiome, or the sponge host. Given that Cyanobacteria regulate the activity of non-photosensitive heterotrophs in planktonic microbial communities of the North Pacific [15], we propose that a similar metabolite-mediated cross-feeding mechanism may underlie the diel transcriptional patterns observed in sponge-associated Alphaproteobacteria of *A. aerophoba*. In addition, Alphaproteobacteria may respond to diel fluctuations in sponge’s internal environment driven by photosymbiont activity – including elevated daytime oxidative stress – rather than only to cyanobacterial metabolites *per se*. Their role in mitigating such stress is supported by their involvement in sulfur-based detoxification pathways, as discussed in the sulfur cycling section below.

### Activity of microbial nitrifiers

Nitrite-oxidizing bacteria (NOB) were detected in our 16S rRNA surveys (Supplementary Fig. S1). Examination of ASVs sequences associated with these lineages revealed close similarity to 16S rRNA genes previously recovered from marine-sponges (Additional File 1: Table S9). Given the well documented photoinhibition of microbial nitrification [75, 76], we assessed differential gene expression in bins annotated as Nitrospinae (bins 75 and 107) and Nitrospirae (bin103) (Fig. 1). No statistically significant differences in gene expression were observed across light-dark models for these NOB lineages. The constitutive expression was unexpected, as light typically inhibits nitrification by damaging enzymatic redox centers [77] and may also impair transcription [78], likely through reactive oxygen species affecting genes and/or transcriptional machinery. However, studies in natural environments indicate that factors such as turbidity and substrate availability can mitigate photoinhibition of ammonium and nitrite oxidation [79]. Although our metatranscriptomic data cannot resolve light-driven impacts on enzyme activity, the consistent expression of NOB genes under both light and dark conditions suggests that transcriptional photoinhibition may be reduced within the holobiont, potentially buffered by photoprotective pigments produced by other sponge symbionts [21].

### Microbial sulfur cycling

Sulfur cycling by microbial symbionts of sponges has been reported in several previous studies [80–82]. *A. aerophoba* is known to harbor anaerobic sulfate-reducing bacteria (SRBs) [26, 83] which can contribute hydrogen sulfide (H_2_S) to the holobiont environment. In our study, the sponge host *A. aerophoba* exhibited upregulation of genes involved in H_2_S detoxification and putative sulfur transport during daylight hours, suggesting potential microbial sulfide production even when photosynthetic cyanobacteria are generating oxygen. However, cyanobacteria are restricted to the outer sponge layer, and additional physiological processes can create anoxic conditions within the sponge regardless of time of day [84–86], supporting the possibility of SRB activity during light periods.

DsrAB are functional genes involved in sulfur reduction, however, they can also act in sulfur oxidation. The direction (oxidation or reduction) of *dsrAB* genes can be determined by phylogenetic affiliation [87, 88]. We performed a phylogenetic analysis of DsrAB transcripts of sponge-associated sulfur cycling lineages from *A. aerophoba* including in the analysis: (i) *dsrAB* transcripts (*n* = 24) from our metatranscriptomes, (ii) *dsrAB* genes (*n* = 4) from the analyzed MAGs, and (iii) a public collection of diverse bacterial and archaeal *dsrAB* [87]. Based on the phylogeny obtained, we identified 23 DsrAB transcripts and 4 *dsrAB* genes predicted to function in sulfur reduction, and only one transcript (affiliated to Gammaproteobacteria) in sulfur oxidation (Supplementary Fig. S7A & B). Two Chloroflexi MAGs (bin90 and 127) showed particularly high levels of *dsrA* expression (Supplementary Fig. S7C&E). We also tested for potential diel periodicity for these transcripts, yet the genes were constitutively expressed at the four time points (Supplementary Fig. S7 C-F). Overall, these data provide evidence for the SRB symbionts being a potential continuous source of H₂S to the sponge environment. However, sulfite – the substrate for dissimilatory sulfite reduction via *dsrAB* - may be more available during daytime, given the observed upregulation of taurine dioxygenase (*tauD*) in the symbiotic alphaproteobacterium bin65 (Additional File 1: Table S7), and the fact that taurine is a widespread sulfonate metabolite in marine sponges [5].

An additional source of H_2_S in the sponge is the degradation of cysteine, which is generated from homocysteine via the transsulfuration pathway and requires vitamin B_6_ as a cofactor. Interestingly, pyridoxal kinase, essential for vitamin B_6_ biosynthesis, was upregulated in the alphaproteobacterial bin65, supporting enhanced cysteine production from homocysteine during daytime. Homocysteine itself is generated from S-adenosylhomocysteine (SAH) by adenosylhomocysteinase (AHCY), and this gene was also upregulated during the day, with its best taxonomic annotation affiliated to the Desulfurellaceae (phylum Desulfobacterota), which are also known to occur as symbionts of *A. aerophoba* [48].

The production of SAH depends on the availability of S-adenosylmethionine (SAM), which is synthesized from methionine. Methionine itself can be produced through multiple pathways, one via MetH, which requires vitamin B_12_, and another via betaine-homocysteine methyltransferase (BHMT), which is B_12_-independent and uses betaine as a methyl donor to convert homocysteine into methionine. Notably, both B_12_ import (alphaproteobacterial bin65) and B_12_ biosynthesis genes (alphaproteobacterial bins 65, 95 and 129) were upregulated during daytime, while betaine availability may arise from choline degradation. Interestingly, choline dehydrogenase from the alphaproteobacterial symbiont bin95 was also upregulated during the day (Additional File 1: Table S7).

H_2_S accumulating in the sponge host from cysteine degradation and sulfite reduction is likely detoxified by the sponge via sulfide: quinone oxidoreductase (SQOR), which was also upregulated during the day (see section on differential expression of host genes). Additionally, during daylight hours, when photosynthesis by *Ca.* S. spongiarum takes place, and oxidative stress within the holobiont is elevated, both the sponge host and its symbionts engaged glutathione-based detoxification pathways: specifically, the sponge through hydroxyacylglutathione hydrolase (Glo2) activity, and the alphaproteobacterial bin65 via glutathione S-transferase (GstB) activity. Expression of both genes increased during the day (Additional File 1: Table S7 and S10).

Taken together, our results indicate that alphaproteobacterial symbionts, sulfate reducing bacteria, and the sponge host are metabolically interconnected through the sulfur cycle, jointly contributing to H_2_S detoxification and glutathione-based oxidative stress defenses during daytime.

### Differential expression of host genes

**Fig. 6:**
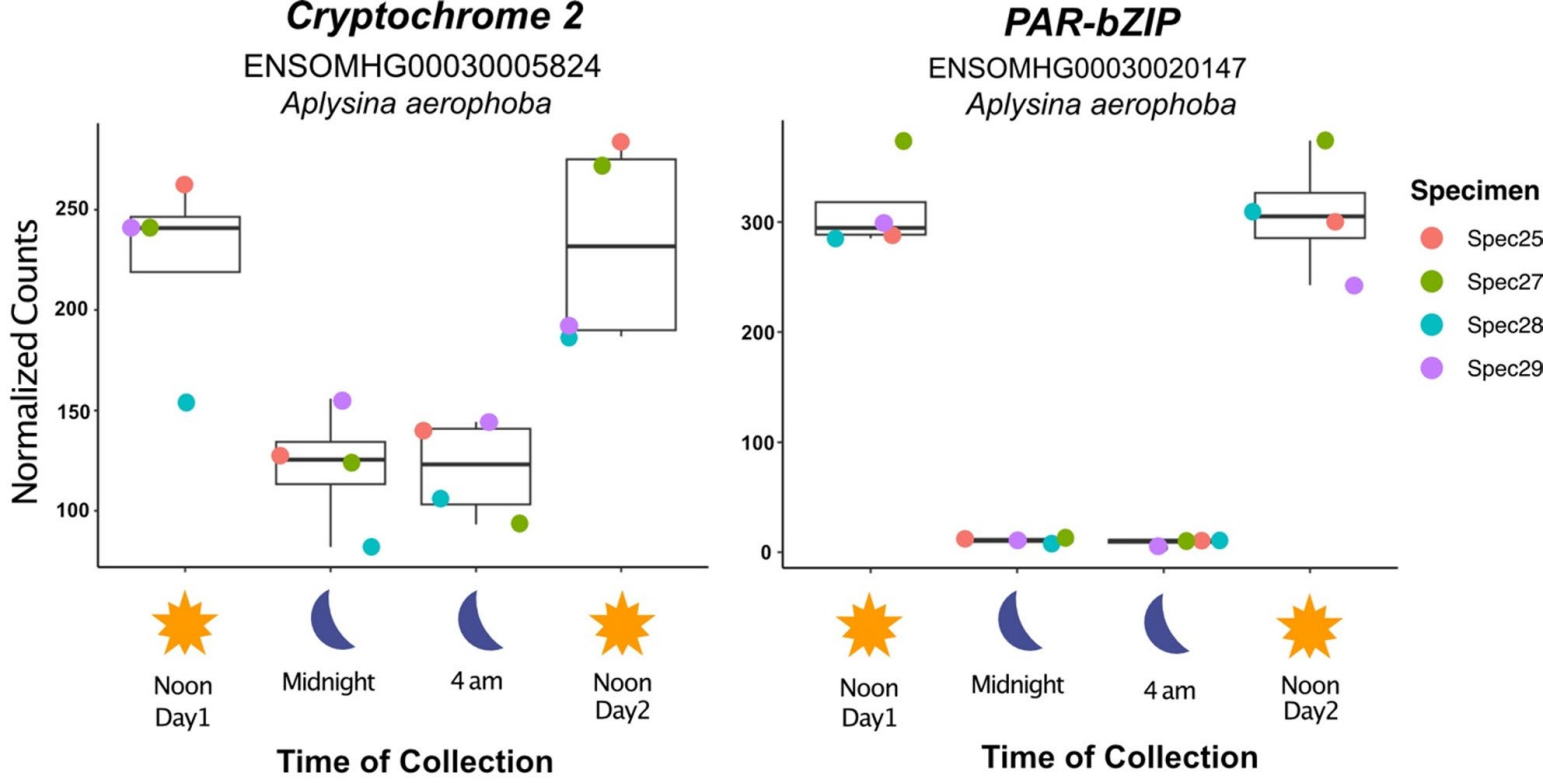
Sponge circadian genes upregulated during the day versus night. Data points, summarized with boxplot quartiles, show normalized expression values for biological replicates color coded as a function of specimen source for each time point (Noon Day1, Midnight, 4 AM, Noon Day2) for genes *cry2* and *PAR-bZIP*, which were significantly upregulated at day *versus* night

In addition to exploring diel-related changes in microbial activity, we also investigated the effect of light availability on the transcriptional profiles of the host sponge *A. aerophoba*. Non-metric multidimensional scaling (NMDS) of normalized read counts revealed no clustering by sampling time or individual sponge specimen (Supplementary Fig. S8). However, differential gene expression analysis using DESeq2 identified orthologs of genes *PAR-bZIP* and *cry2* as the only circadian genes exhibiting a clear diurnal pattern (Additional File 1: Table S10). In all four specimens examined, transcript levels of both *PAR-bZIP* and *cry2* were elevated at noon compared to midnight and 4 AM (Fig. 6). This observation aligns with findings in the sponge *Amphimedon queenslandica*, where these two genes were also the only circadian genes displaying diel expression, supporting the hypothesis that the circadian clock of ancestral metazoans was simpler than that of eumetazoans [12].

Additional host transcripts enriched during the light period included genes related to multidrug resistance, detoxification via active export, and antioxidant activity (Supplementary Fig. S9), suggesting that daytime conditions may lead to increased oxidative stress and the buildup of toxic metabolites. This daytime stress response in the host reflects similar transcriptional trends in microbial symbionts, such as those affiliated to Candidatus Poribacteriota and Chloroflexota (Dehalococcoidia), which also showed higher expression of multidrug resistance genes and genes involved in carotenoid biosynthesis during daylight hours (Fig. 2).

Two host genes associated with sulfur metabolism - one encoding a YeeE/YedE family membrane protein (TCDB 9.B.102) putative sulfur/selenium transporter and the other involved in hydrogen sulfide (H_2_S) detoxification - were also up-regulated during the day (Supplementary Fig. S10) and are discussed in the previous section on sulfur cycling and oxidative stress detoxification.

Interestingly, some host genes were uniquely up-regulated at 4 AM, including chaperon proteins involved in protein folding (*dnaJ* and heat shock proteins) and a transcriptional factor known to bind CCAAT promoter motifs (Supplementary Fig. S11). The environmental or physiological triggers underlying this nocturnal induction of stress-response genes remain unclear but may reflect transient cellular stress or preparatory responses to upcoming changes in metabolic activity towards dawn. In addition, some genes were upregulated in the night (midnight and 4 AM), including those involved in glycogen catabolism and inositol biosynthesis (Supplementary Fig. S12), pointing to coordinated changes in energy management and inositol-based signaling during the dark period. This suggests that *A. aerophoba* modulates its metabolic and signaling pathways in a time-of-day-dependent manner, potentially in response to reduced photosynthate availability or changes in microbiome-derived metabolite fluxes.

## Conclusions

Metatranscriptome studies analyzing both host and microbial gene expression in mutualisms remain rare, especially in naturally occurring animal systems that reflect how these associations function in situ. Here, we present the first such analysis of diel transcriptional dynamics in a marine sponge holobiont. While the overall structure and activity of the *A. aerophoba* microbiome remained stable across time points and individuals, distinct diel transcriptional patterns were observed in specific microbial lineages and in the host sponge. Cyanobacterial photosymbionts exhibited classic circadian regulation, with daytime enrichment of genes related to light-driven processes, while also showing nighttime upregulation of glutamine synthase and ammonia channels, suggesting that host-derived carbohydrates may fuel nitrogen assimilation in the dark – a pattern that contrasts with free-living cyanobacteria. Specific heterotrophic Alphaproteobacteria also showed diel expression, likely driven by light-mediated environmental or metabolic shifts, rather than intrinsic circadian control. By contrast, most sponge-associated microbes did not display diel expression patterns, suggesting that most microbes are not directly synchronized to light-driven processes. The sponge host displayed diel regulation of circadian genes and genes linked to oxidative stress, detoxification, and sulfur metabolism, patterns mirrored by microbial taxa that contribute to sulfur cycling and detoxification. These results reveal not only how sponges and their symbionts respond to daily environmental fluctuations, but also how metabolic interactions – particularly via sulfur-based pathways and photoprotective pigment biosynthesis – coordinate detoxification and stress mitigation, providing new insight into the stability and functional integration of one of the oldest animal-microbe partnerships.

## Supplementary Information

Below is the link to the electronic supplementary material.

Supplementary Material 1

Supplementary Material 2

## Data Availability

All metatranscriptomes used in this study are publicly available under NCBI BioProject PRJNA1256915, with accession numbers SAMN48207605-SAMN48207668.

## Abbreviations

cry2: Cryptochrome 2
MRN: Median Ratio Normalization
NMDS: Non–metric multidimensional scaling
nr: non–redundant
ORF: Open reading frame
PAR-bZIP: Proline and Acidic amino acid–Rich basic Leucine Zipper protein
PETs: Protein encoding transcripts
pHMMs: profile Hidden Markov Models
QC: Quality control
RND: Resistance–Nodulation–Division
ROS: Reactive oxygen species
TPM: Transcripts per Million
AOA: Ammonium Oxidizing Archaea
NOB: Nitrite Oxidizing Bacteria
MAG: Metagenome Assembled Genome
SRB: Sulfur Reducing Bacteria
