# Supplementary material for: Diel transcriptional dynamics of a marine sponge and its microbiome in a natural environment
Source: Anim Microbiome. 2026 Feb 5;8:12. doi: 10.1186/s42523-025-00510-z (PMC12879404; doi:10.1186/s42523-025-00510-z)
Supplement: Supplementary file 1 — Supplementary Material 1 [file 42523_2025_510_MOESM1_ESM.docx]

*Supplemental Materials*

**Diel transcriptional dynamics of a marine sponge and its microbiome in a natural environment**

Gustavo A. Ramírez^1,2^*, Rinat Bar-Shalom^1^, Tzipora Perez^1^, Reut Efrati Epchtien^1^, Andrea Furlan^1^, Roberto Romeo^3^, Michelle Gavagnin^1^, Arkadiy I. Garber^4^, Maya Lalzar^5^, Laura Steindler^1^*.

^1^ Department of Marine Biology, Leon H. Charney School of Marine Sciences, University of Haifa, Israel.

^2^ Department of Biological Sciences, California State University, Los Angeles, CA, USA.

^3^ Istituto Nazionale di Oceanografia e di Geofisica Sperimentale (OGS), Trieste, Italy.

^4^ School of Life Science, Arizona State University, Tempe, AZ, USA.

ORCIDs - GAR: 0000-0001-8122-4898, RBS: 0000-0001-8115-2388, TP: 0009-0007-2764-936X, RR: 0000-0001-5867-8998, AIG: 0000-0001-7935-0246, ML: 0000-0001-8386-3490, LS: 0000-0002-0188-101X

*Corresponding Authors:

Laura Steindler

Department of Marine Biology

Leon H. Charney School of Marine Sciences

University of Haifa, 199 Aba Khoushy Ave

Mount Carmel, Haifa, Israel

Email: [lsteindler@univ.haifa.ac.il](mailto:lsteindler@univ.haifa.ac.il)

Tel. +9724-8288987; Fax +9724-8288267

Gustavo A. Ramírez

Department of Biological Sciences

California State University, Los Angeles

5151 State University Drive

Los Angeles, CA, 90032

Email: [gramir157@calstatela.edu](mailto:gramir157@calstatela.edu)

Tel: +01 323-343-7480


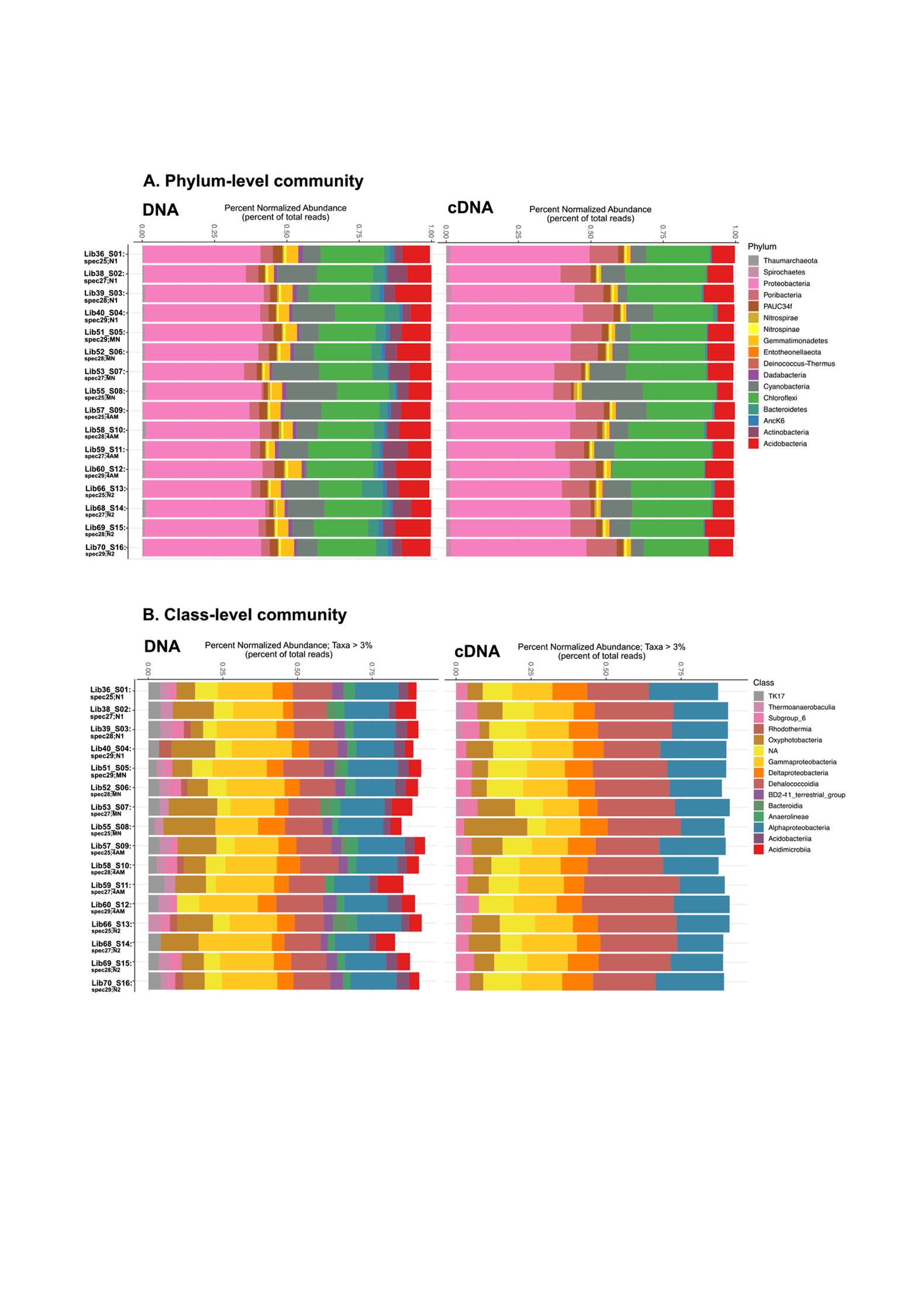


**Figure S1:** Community composition based on 16S rRNA genes and transcripts Amplicon Sequence Variants (ASVs), at the (A) Phylum and (B) Class taxonomy levels, recovered from each library in this study. The following libraries, sampled from four individual sponges (see spec. number on the row legend) represent the following time of sample collection for both DNA and cDNA: Noon1: 36, 38, 39, and 40; Midnight: 51, 52, 53, and 55; 4AM: 57, 58, 59, 60; Noon2: 66, 68, 69, 70.

**Figure S2:** Transcriptional profiles of representative Chloroflexi (A, B) and Poribacteriota (C, D) dominant community members. Statistical models (PERMANOVA performed on the original Bray-Curtis distance matrix, alpha = 0.05) do not show significant differences based on Daytime vs. Nighttime.

**Figure S3:** Poribacteria Bin 44 DE genes: Day versus Night model.

**Figure S4:** Differential abundance testing for (A) Alphaproteobacteria Bin65 and (B) Cyanobacteria Bin9. Clockwise results are depicted for the following comparisons Noon1 vs. Noon2, Combined Noon timepoints vs. Midnight, and Combined Noon timepoints vs. 4am. Significantly differentially enriched PETs are only detected for the Bin9 Combined Noon vs. Midnight and 4AM comparisons depicted in panel B. Results for all significantly enriched PETs are summarized in a radar plot. Results for PETs significantly enriched in darkness (either 4AM or midnight) are labeled in purple font and shown in the inner purple circle in negative TPM log2 fold values. Conversely, PETs significantly enriched in daytime samples are labeled in yellow font and depicted in the outer yellow circle in positive TPM log2 fold values.


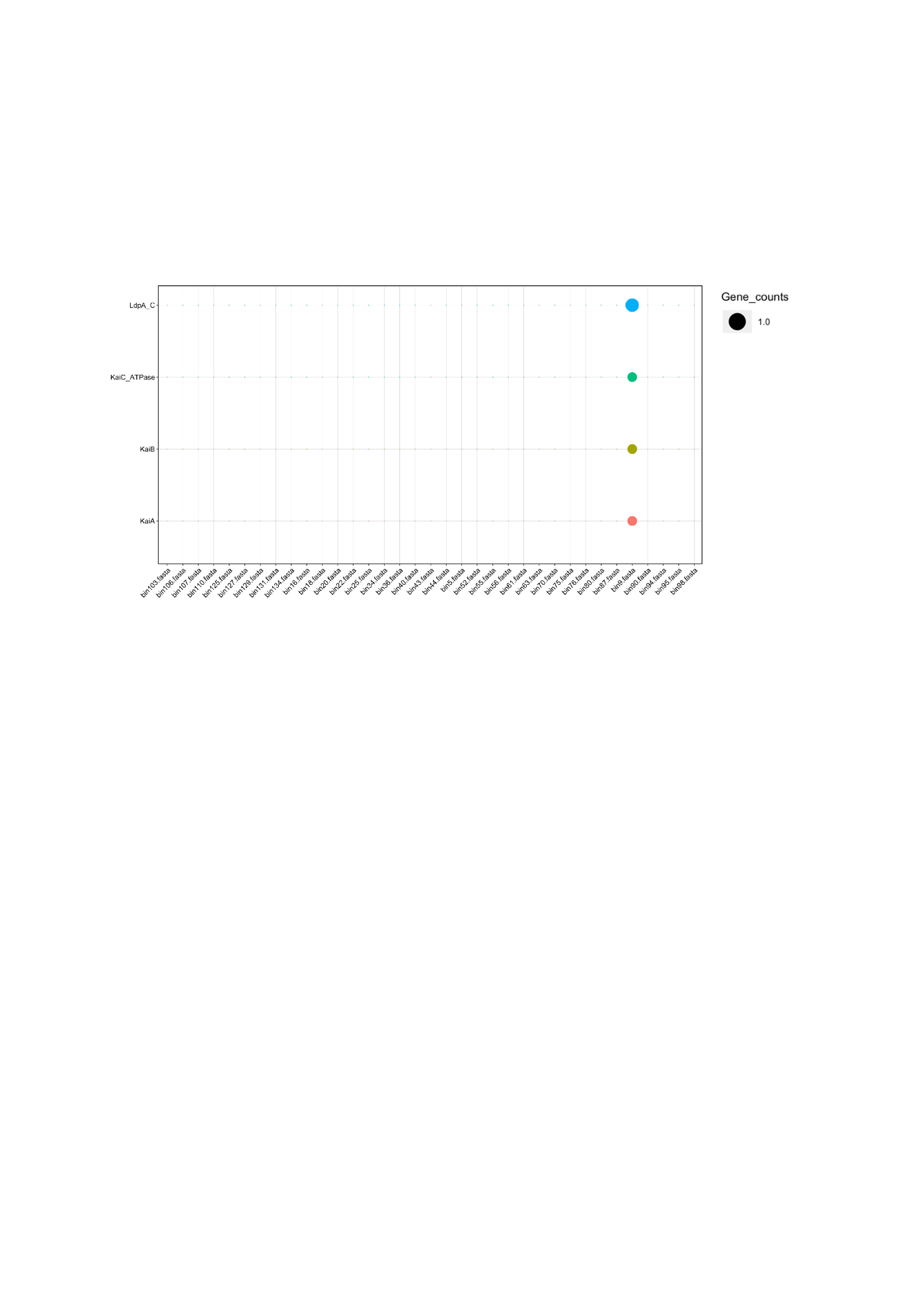


**Figure S5:**

Profile Hidden Markov Model-based survey of circadian clock-related protein homologues across all bins included in this study. Search was implemented using pHMMs downloaded from Pfam and run on a custom script, *CircGenie*, available in the Magic Lamp suite (available here: https://github.com/Arkadiy-Garber/MagicLamp) customized for this study specifically.

**Figure S6. Differentially expressed genes for the three Alphaproteobacteria bins exhibiting light-driven transcriptional patterns.** A) For each MAG, differentially expressed protein statistics are summarized in Venn diagrams with each circle representing time of collection (Noon, midnight (MN), and 4AM). B. Frequency-weighted word cloud summaries for combined Alphaproteobacterial proteins associated with time of collection; in other words, the largest words represent the most common annotations for all proteins collected at each timepoint. C) Differentially expressed genes associated with nucleic acid repair. D) Differentially expressed genes associated with Vitamin B12 production. E) Differentially expressed genes associated with other metabolisms.

**Figure S7:** Phylogenetic analysis of DsrAB amino acid sequences. A) DsrAB phylogenetic tree including homologous metatranscriptome PETs. B) Tree branch highlighting the placement of DsrAB transcripts from MAGs. C-F) Expression of *dsrAB* through the diel cycle, in four MAGs.


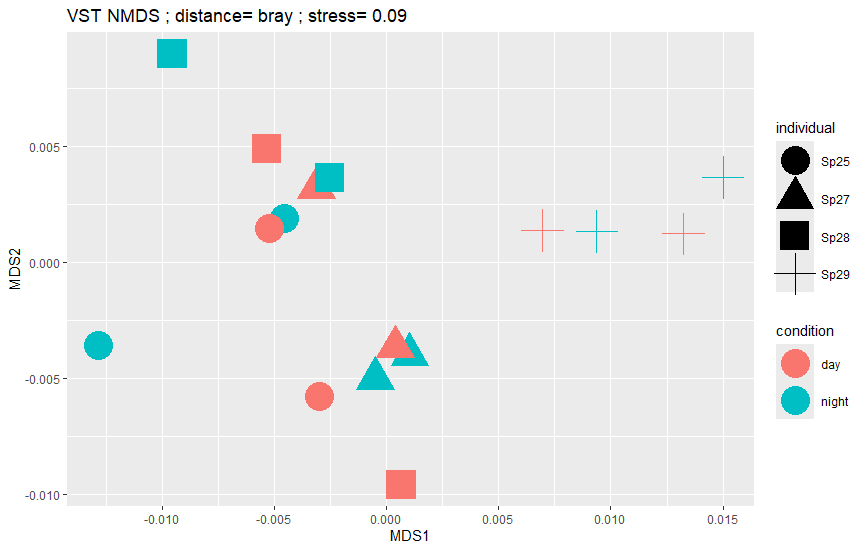


**Figure S8:**

NMDS plot of normalized read counts (output of DESeq2) show no clustering of host transcripts according to condition (day/night) nor sponge subject (individual).


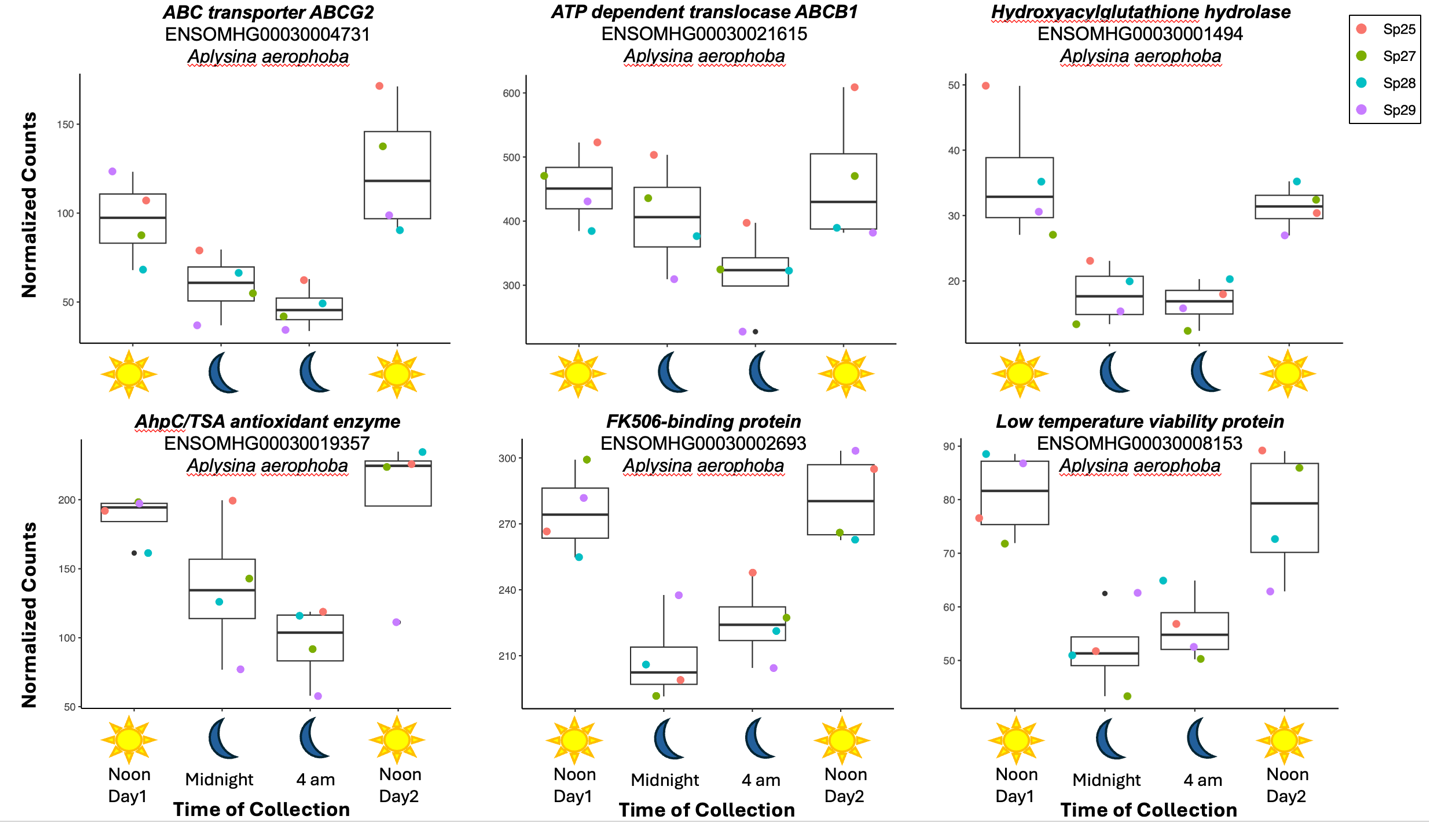


**Figure S9:**

Sponge genes involved in detoxification, multidrug resistance and protein stability upregulated during the day versus night. Data points, summarized with boxplot quartiles, show normalized expression values for biological replicates color coded as a function of specimen source for each time point (Noon Day1, Midnight, 4am, Noon Day2).


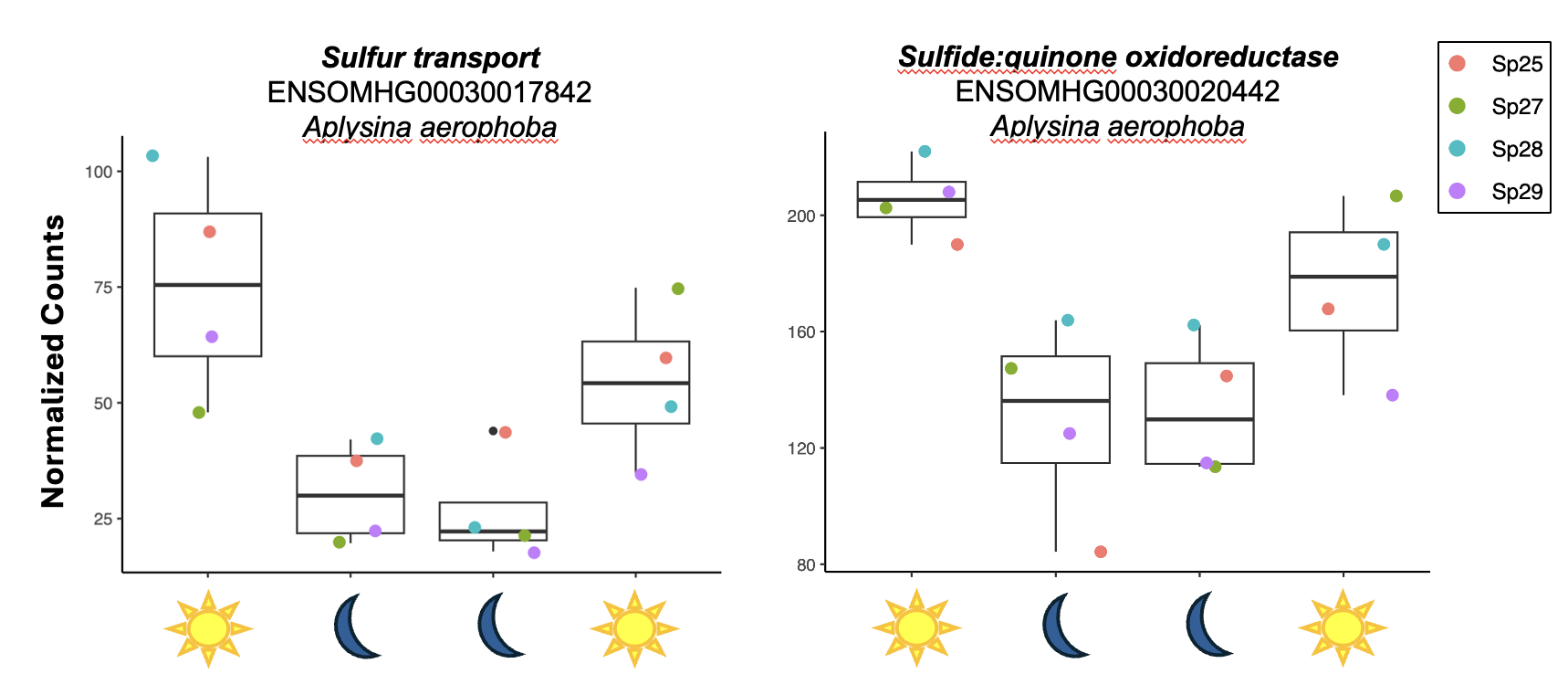


**Figure S10.** Sponge genes involved in putative sulfur transport and sulfide detoxification, upregulated during the day versus night. Data points, summarized with boxplot quartiles, show normalized expression values for biological replicates color coded as a function of specimen source for each time point (Noon Day1, Midnight, 4 AM, Noon Day2).


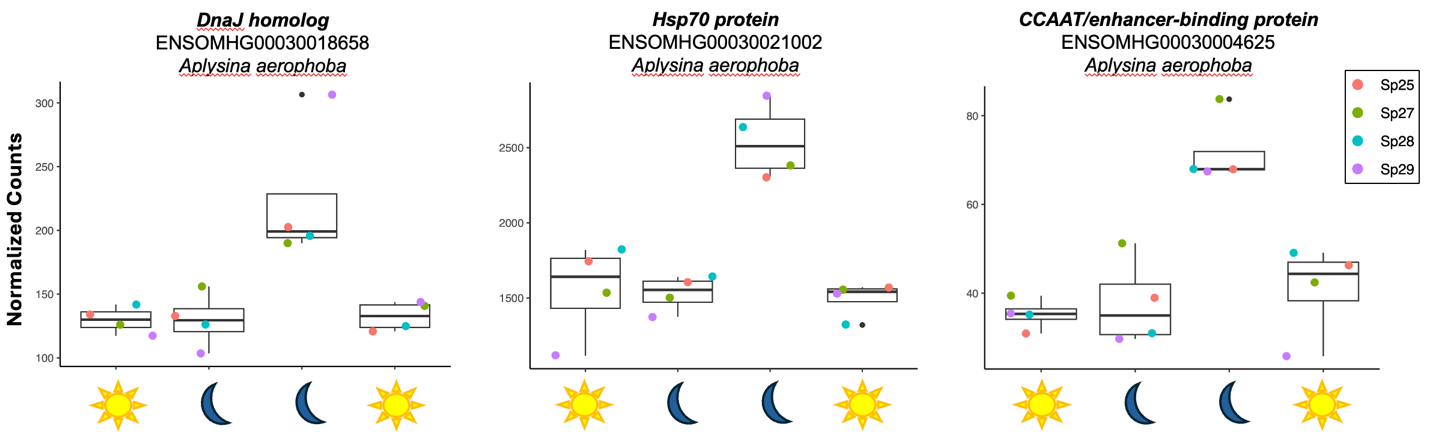


**Figure S11.** Sponge genes involved in protein folding and transcriptional regulation, upregulated at 4 AM. Data points, summarized with boxplot quartiles, show normalized expression values for biological replicates color coded as a function of specimen source for each time point (Noon Day1, Midnight, 4 AM, Noon Day2).


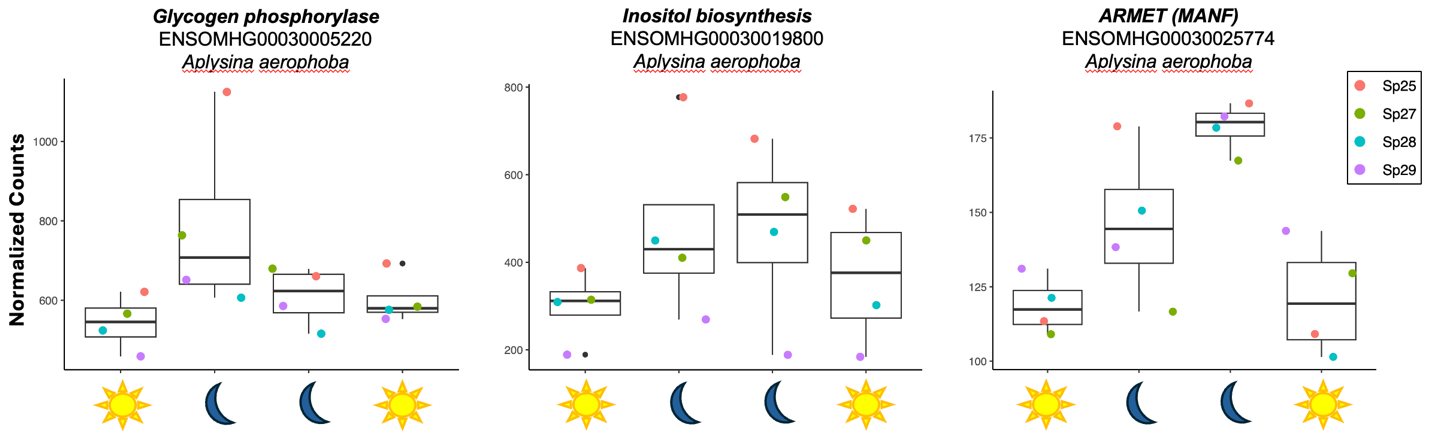


**Figure S12.** Sponge genes involved in energy metabolism and signaling pathways, upregulated at night. Data points, summarized with boxplot quartiles, show normalized expression values for biological replicates color coded as a function of specimen source for each time point (Noon Day1, Midnight, 4am, Noon Day2).

**Extended Materials and Methods**

*Methods – CircGenie development*

Identification of genes for cyanobacterial circadian rhythm was carried out using a custom software tool, CircGenie, freely available under the following GitHub repository: <https://github.com/Arkadiy-Garber/MagicLamp>. CircGenie carries out protein prediction using Prodigal ^1^, and then queries, using hmmsearch from the HMMER software package ^2^, profile hidden Markov models against protein sequences predicted from genomes or metagenome-assembled genomes (MAGs) of interest. CircGenie features seven HMMs, collected from the Pfam database of protein families. CircGenie reports all protein matches surpassing the Pfam-designated bitscore cutoff, with the exception of the genes that are part of the *kaiABC* operon. In order to be reported, at least two of the three genes that are part of this operon need to be present and adjacently encoded on the genome.

1 Hyatt, D. *et al.* Prodigal- prokaryotic gene recognition and translation initiation site identification. *BMC Bioinformatics* **11** (2010).

2 Johnson, L., Eddy, S. & Portugaly, E. Hidden Markov model speed heuristic and iterative HMM search procedure. *BMC Bioinformatics* **11**, doi:<http://www.biomedcentral.com/1471-2105/11/431> (2010).

**Supplemental Files**

Table S1. Sequence retention following quality control based on sequence quality, adapter trimming, and ribodepletion steps. Total sequence retention ranged from 40-55% of initial sequence counts with a mean average of 46.34% for all metatranscriptomic libraries.

Table S2. Total number of reads from each sample that were mapped to *A. aerophoba.*

Table S3. Differentially abundant transcripts using global (all libraries) day vs. night model.

Table S4: Forty-seven non-redundant DE PETs from Noon vs. 4AM and Noon vs. Midnight models and DE stats (50 instances with redundancy).

Table S5: BIN44:Poribacteria DE Day vs Night Model.

Table S6: Cyanobacterial Bin9_DE genes with annotation (used for Figure 5A).

Table S7: Differentially abundant transcripts for Alphaproteobacteria bin65, 95, and 129, comparing noon-selected samples with midnight- and 4 AM- collected samples independently.

Table S8: Alphaproteobacterial DE "hypothetical" protein analysis.

Table S9: Sequence taxonomy (SILVA138) for dominant AOA _AOB_NOV ASVs.

Table S10: Differential expression of host genes
